# Tailoring Wettability Properties of GaN Epitaxial Layers through Surface Porosity Induced during CVD Deposition

**DOI:** 10.1021/acs.langmuir.1c02316

**Published:** 2021-12-10

**Authors:** Josué Mena, Joan J. Carvajal, Vitaly Zubialevich, Peter J. Parbrook, Francesc Díaz, Magdalena Aguiló

**Affiliations:** † Física i Cristal·lografia de Materials i Nanomaterials (FiCMA-FiCNA) and EMaS, Departament Química Física i Inorgànica, Universitat Rovira i Virgili (URV), Tarragona 43007, Spain; ‡ Department of Physics, Umeå University, Umeå SE-90187, Sweden; § Tyndall National Institute, Lee Maltings, Cork T12 R5CP, Ireland; ∥ School of Engineering, University College Cork, Cork T12 R5CP, Ireland

## Abstract

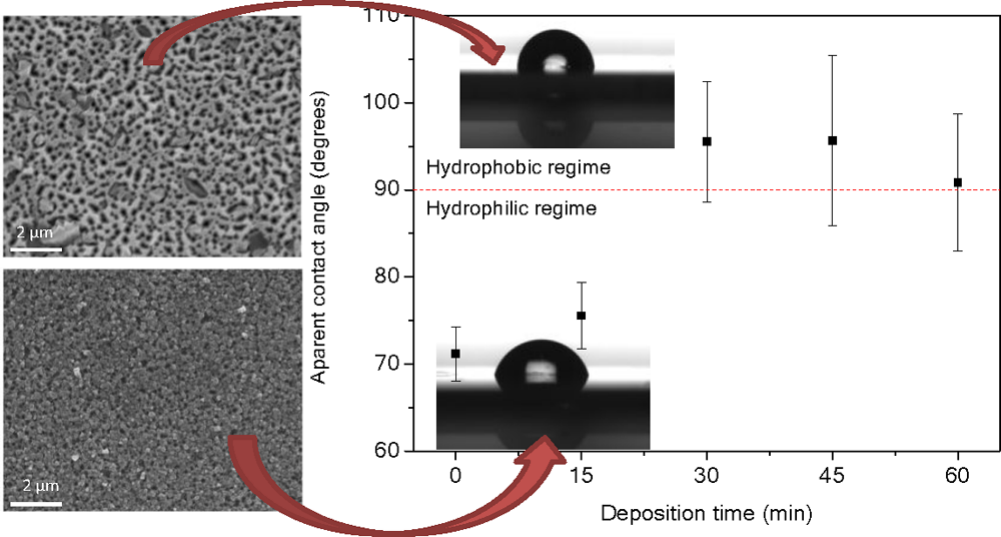

Porous GaN epitaxial layers were prepared using single-step chemical vapor deposition (CVD) through the direct reaction of ammonia with gallium. The degree of porosity and pore diameters in the resulting GaN were analyzed by means of SEM and AFM and were found to depend on the GaN deposition time. Furthermore, the evolution of the contact angle of a droplet of water located on the surface of these GaN epitaxial layers with the deposition time was investigated. We observe a transition from the hydrophilic regime to the hydrophobic regime for deposition times longer than 15 min. The observed dependence of GaN hydrophobicity on its degree of porosity is discussed and explained in the framework of the Cassie–Baxter model.

## 1 Introduction

GaN is a semiconductor that is well known for its applications in electronics and optoelectronics because of its wide band gap, large critical electric field, high electron mobility, and reasonably good thermal conductivity and stability.^1^ All of these properties make GaN an excellent candidate for the fabrication of commercial high electron mobility transistors (HEMTs),^2^ light-emitting diodes (LEDs),^3^ and laser diodes (LDs),^4^ among others.

In the past decade, many prototypes of chemical and biochemical sensors have been also developed on the basis of GaN HEMT devices^5−8^ since GaN offers great chemical stability^9^ that is essential for sensing in aqueous media. Thus, sensors based on GaN that are able to detect hydrogen, carbon monoxide, ethylene, acetylene, nitrous oxide, combustion gases, pH, polar liquids, block copolymers, pressure, and biological species such as prostate-specific antigen have been developed.^5−8^ This allows for the development of integrated chemical gas and fluid monitoring sensors compatible with high bit-rate wireless communication systems that facilitate their use in remote arrays.^6,7^

For this reason, it is desirable to control the wettability of the exposed surface of the sensing devices to the samples, biological and chemical, so that no impurities get attached to the surface, favoring the obtaining of a reproducible sensor. To change the wetting properties of a surface, two different strategies can be used: (i) its functionalization with polar molecules to make it more hydrophilic or apolar molecules to make it more hydrophobic^10^ and (ii) roughening it, making an already hydrophilic surface even more hydrophilic if it follows the Wenzel model^11^ or more hydrophobic if it follows the Cassie–Baxter model.^12^ Also, by roughening the surface through the generation of pores, the surface area of the device increases, as does the area of the contact surface against which the analytes can interact, allowing an increase in the sensitivity of the sensors.^5^

Porous GaN was produced first through anodization in an aqueous solution of HF under UV illumination to be used as a buffer layer for heteroepitaxial growth on foreign lattice mismatched substrates since the porous structure allows for the relaxation of the structure.^13^ Since then, many other top-down approaches have been developed to induce porosity in GaN layers, such as photoelectrochemical wet etching,^14^ metal-assisted electroless etching,^15^ and alternating current photoassisted electrochemical etching.^16^ Aside from its utility as a buffer layer, the porous form of GaN has physical properties that differentiate it from bulk GaN, such as a strong photoresponse,^17^ a UV shift of the band gap due to quantum confinement if the width of the walls between pores is small enough,^18^ a photoluminescence intensity enhancement,^14^ and a high luminescence extraction efficiency.^19^ Porous GaN can also be produced by bottom-up approaches such as the direct reaction of metallic Ga and NH_3_ in CVD systems.^20^

In this work, we studied the influence of surface porosity on the apparent contact angle of a sessile drop of water deposited on the top of the surface of porous GaN layers produced by chemical vapor deposition (CVD). We also analyzed how the different pore sizes, tuned by the deposition time, affect the wetting properties of the material.

## 2 Experimental Section

### 2.1 Deposition of Porous GaN Films by CVD

Porous GaN films were deposited epitaxially on substrates composed of a 1-μm-thick p-type GaN(0001) doped with Mg/3-μm-thick undoped GaN(0001)/sapphire(0001). We used the direct reaction between metallic Ga and NH_3_ in a CVD 2 in. Thermolyne 79300 horizontal tubular furnace according to the procedure optimized previously.^20^ The substrate was placed facing downward on a boron nitride (BN) support ∼1.7 cm above the Ga source. The quartz tube reactor was degassed to a pressure below 10^–2^ Torr. Ammonia was then introduced into the quartz tube reactor via a mass-flow controller at a preset flow rate of 75 sccm. The pressure was kept at 15 Torr while the furnace was heated to 1203 K and maintained at this temperature for four different deposition times, 15, 30, 45, and 60 min, under a constant flow of NH_3_. By changing the deposition time, pores of different sizes could be produced.^20^ The deposition of GaN was stopped by closing the NH_3_ flow and turning off the furnace heating system, allowing it to cool to room temperature under a pressure of 10^–2^ Torr.

### 2.2 Morphological Characterization of the Porous GaN Films

The surface morphology of the nanoporous GaN films was characterized using a JEOL JSM 6400 scanning electron microscope (SEM) and by atomic force microscopy (AFM) with an Agilent 5500 microscope operating in tapping mode, using Si tips with a radius of <10 nm, oscillating at a resonance frequency of 75 kHz.

### 2.3 Measurement of the Apparent Contact Angle

To evaluate the apparent contact angle (ACA) on the different GaN samples, a 2 μL water droplet was placed on the top of the GaN surface smoothly with a micropipette. The droplet profile was recorded using an OCA 15EC video-based optical contact angle measuring goniometer by NEURTEK Instruments.

## 3 Results and Discussion

### 3.1 Morphological and Topographical Characterization of the Porous GaN Films Produced by CVD

**Figure 1:**
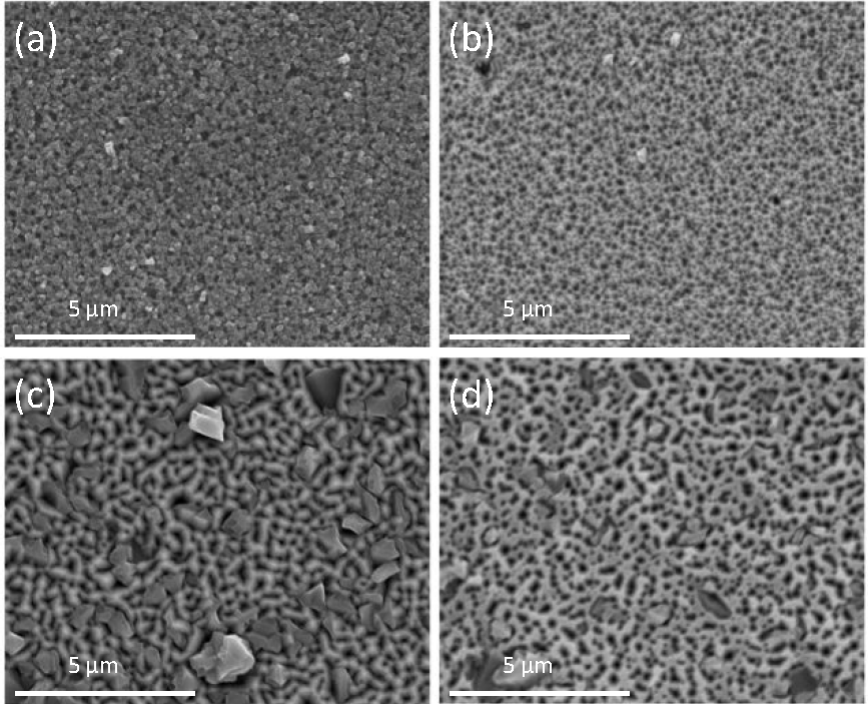
SEM images of the surface of porous GaN layers produced by CVD grown for (a) 15, (b) 30, (c) 45, and (d) 60 min.

The surface morphology of the porous GaN samples was characterized using SEM. Figure 1 shows top-view SEM images of the porous GaN samples grown at different deposition times. The images reveal that samples grown for shorter times show more circularly shaped pores with apparently smaller sizes, while in samples grown for longer times, the pores present a more diverse range of shapes. In all cases, the pores observed in the grown layers are aligned along the [0001] direction, perpendicular to the substrate, matching the crystallographic orientation of the substrate. It is worth noting that GaN particles^21^ also appear during the deposition of the porous GaN layer in some cases. From Figure 1c,d, we can deduce that the nucleation of the particles occurs in the early stage of the growth process since the porous epitaxial layer encircles the particles as the growth proceeds.

**Figure 2:**
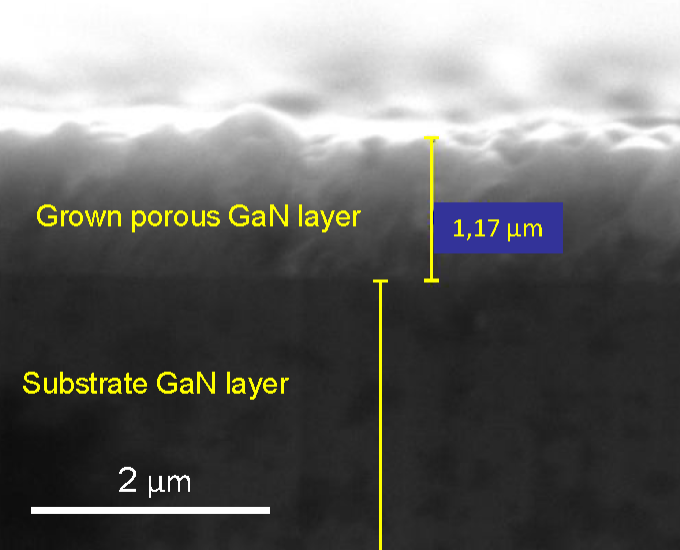
Cross-sectional SEM image of the porous GaN sample grown for 30 min showing the thickness of the layer and the pore depth.

Figure 2 shows a cross-sectional SEM image of the sample grown for 30 min. A sharp interface divides the initial GaN substrate and the grown porous GaN layer. The change in color observed in the image reveals the change in the density of the sample due to the porosity induced in the sample compared to that of the nonporous GaN substrate. This image reveals that the deposited GaN layer grown for 30 min has a thickness of ∼1.17 μm. The evolution of the layer thickness with time was previously reported by analyzing the cross-sectional images for samples grown at different times, showing an increase in the thickness with a deposition time of up to ∼1.7 μm for the sample grown for 60 min.^20^

**Figure 3:**
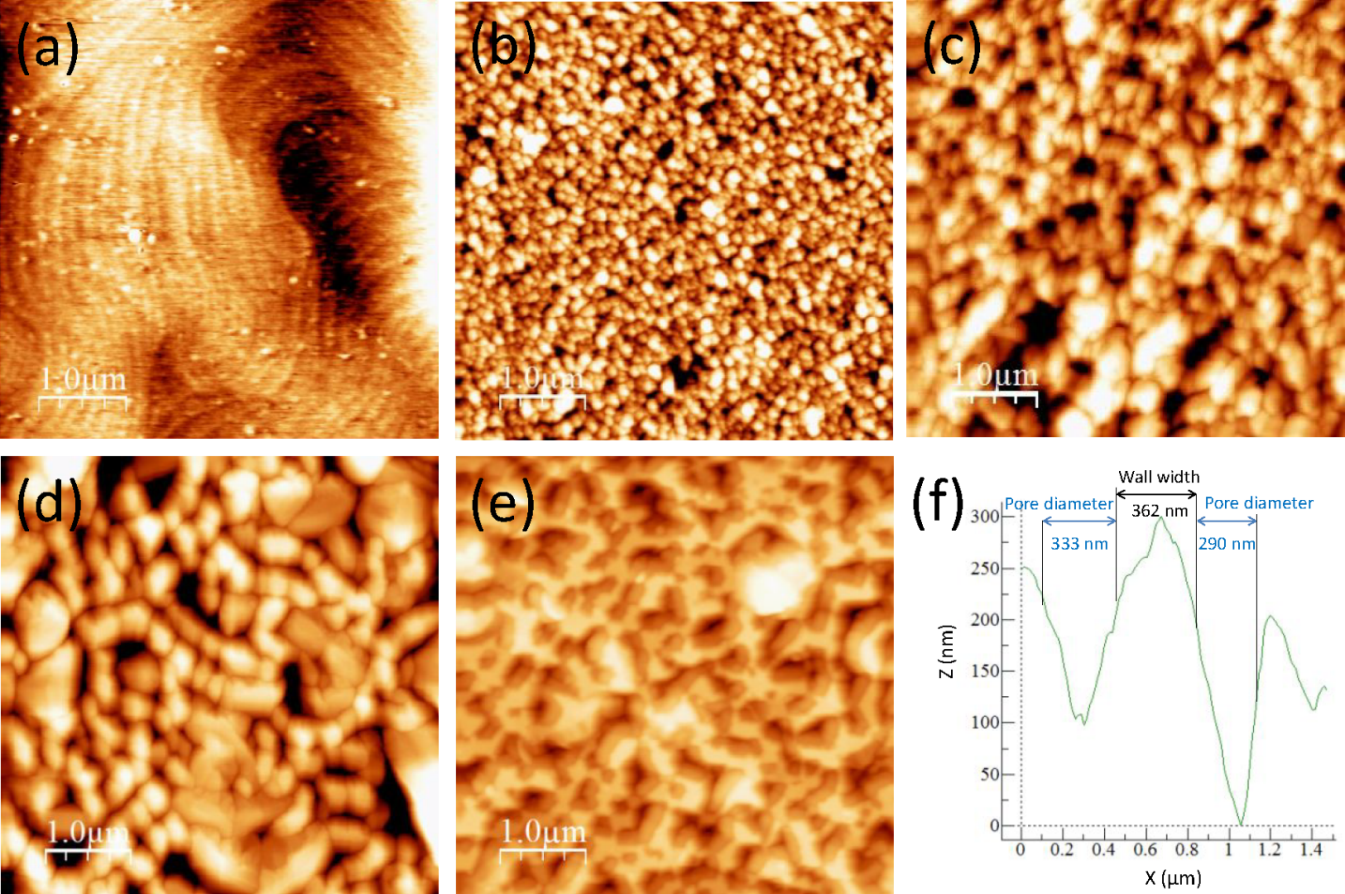
AFM images of the surface of (a) a GaN substrate and CVD porous GaN layers grown for (b) 15, (c) 30, (d) 45, and (e) 60 min. (f) Criteria used to determine the pore diameters and the wall widths.

The surface topography of the GaN samples was also analyzed by tapping mode AFM. Figure 3a shows an AFM image of a GaN substrate. In it, a flat surface can be seen with steps 190 nm wide and a terrace thickness of ∼5 Å, which matches the GaN unit cell constant *c* (5.18 Å).^22^Figure 3b–e shows the AFM images corresponding to the porous GaN samples grown for different times. The profiles obtained from the AFM images provide an estimated value of the porous diameters and also of the wall thicknesses between pores, determined by considering the distance between the peak and the valley of the profiles. Figure 3f depicts the criteria we used to measure the pore diameters and wall thicknesses between pores from an AFM profile. For each sample, 60 different values of both pore diameters and wall thicknesses were taken into account to establish their mean values. Table 1 summarizes these values and their standard deviations.

**Table 1:** Mean Pore Sizes and Wall Thicknesses for the Porous GaN Epitaxial Layers Grown at Different Deposition Times Determined from the Analysis of the AFM Images

deposition time (min)	mean pore diameter (nm)	wall thickness (nm)
15	113 ± 34	113 ± 31
30	252 ± 68	217 ± 63
45	241 ± 109	278 ± 63
60	226 ± 66	243 ± 72

After the AFM images were observed and the pore diameters and wall thicknesses were determined, one could conclude that the sample grown for 15 min has the smallest pore diameters and wall thicknesses among all of the samples. For higher deposition times, pore diameters increase, reaching a maximum for a deposition time of 30 min, upon which the pore size decreases again. However, the wall thicknesses reach their maximum values for the sample grown during 45 min, for which the presence of particles deposited on the surface could also be observed, which complicated the accurate determination of the pore size. The increase in the wall thicknesses as the deposition time increases can be explained by the coalescence of neighboring pores as the layer thickness increases, favored by the deposition of more material, leading to thicker walls but also wider pores.

The pore diameters obtained through the AFM profiles are similar to those determined using an image-processing method.^23^ The discrepancy between the results obtained by SEM and AFM may arise from the image-processing method used in the first case, which considers circular pores to calculate their areas. However, in the AFM characterization the size of the pores is measured transversely, as indicated in Figure 3f.

### 3.2 Analysis of the Wetting Properties

Considering a flat, rigid, smooth, chemically homogeneous surface, the contact angle at the triple contact point is described by Young’s equation
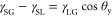
1where *γ*_SG_, *γ*SL, and *γ*_LG_ are the surface tensions of the solid/gas, solid/liquid, and liguid/gas interfaces and *θ*_y_ is the Young contact angle. Macroscopically, this condition is not fulfilled unless the ideal conditions mentioned before are satisfied; however, even if we have a nonflat surface, this condition has to be locally satisfied at every triple contact point.

Surface roughness affects the macroscopic wetting properties of the material, converting a hydrophilic material into a hydrophobic one or into a more hydrophilic one, depending on the wetting properties of the liquid in the microstructure. When the liquid of a droplet is in contact with all of the material’s surface, completely filling the pore voids, the apparent contact angle (ACA) of the droplet is described by the Wenzel model^11^
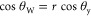
2were *θ*_W_ is the Wenzel contact angle and *r* is the roughness parameter, which is the ratio between the real surface area and the projected flat surface area, a parameter that is always larger than 1. Note that by using eq 2, hydrophobic materials become more hydrophobic by increasing the roughness (*θ*_W_ < *θ*y) while hydrophilic materials become more hydrophilic (*θ*_W_ > *θ*_y_). This model predicts that the microstructure of the surface always amplifies the hydrophilicity of a hydrophilic substrate, so it is valid only when no air is between the droplet and the substrate.

Another wetting mechanism is the so-called Cassie–Baxter model in which the liquid does not completely wet the surface of the material and there is some gas between the liquid and the surface, creating a composite material. This wetting mechanism amplifies the hydrophobicity of both hydrophobic and hydrophilic materials. The transition between the Wenzel model and the Cassie–Baxter model depends on the Young contact angle and the geometric parameters of the rough surface. The equation that describes the Cassie–Baxter contact angle is
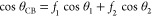
3where cos *θ*_CB_ is the Cassie–Baxter contact angle and *f*_1_ and *f*_2_ are the areal fractions of the solid and the air in contact with the liquid droplet, respectively. The sum of these terms invariably equals 1. *θ*_1_ and *θ*_2_ are the Young contact angles between the solid and liquid and between the air and liquid, respectively. This equation can be rewritten as a function of only *f*_1_ and *θ*_1_ since *θ*_2_ = 180°:^24^
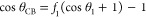
4Thus, when a hydrophilic material becomes hydrophobic after roughening, this situation can be explained only through the formation of a composite interface between the material and the trapped air beneath the water droplet, as described by the Cassie–Baxter model.^12^ Thus, water cannot penetrate the pores because of the resistance of the trapped air inside them.

It is important to consider that the Wenzel and Cassie–Baxter models are valid only when the dimensions of the microstructures on the surface are much smaller than the dimensions of the liquid droplet,^25^ as happens in our case.

**Figure 4:**
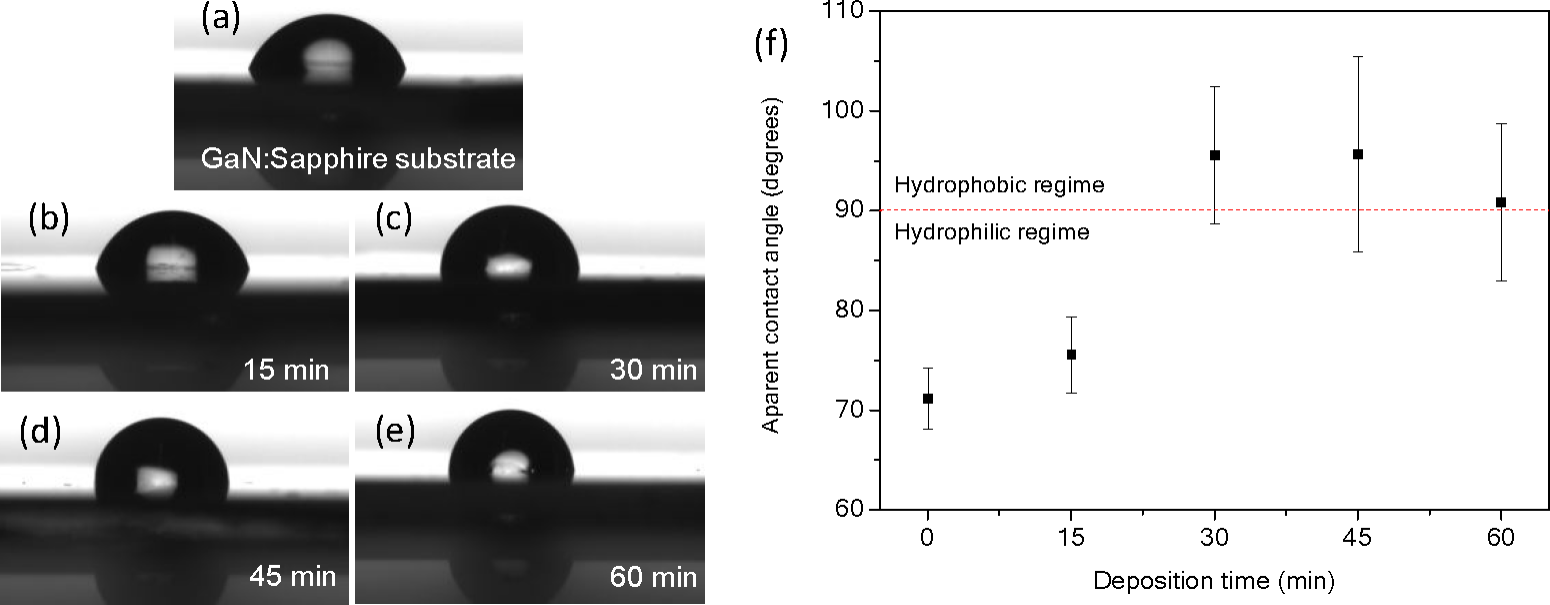
Optical images of the apparent contact angle of a drop of water on (a) a p-type nonporous GaN substrate and CVD porous GaN layers grown for (b) 15, (c) 30, (d) 45, and (e) 60 min. (f) Apparent contact angle as a function of the deposition time.

Images of a water droplet standing on the top of the surface of different porous GaN samples as well as on a nonporous substrate are shown in Figure 4. To determine the ACA, five measurements were taken for each sample. The values of the ACA as a function of the deposition time are shown in Figure 4f. The values of the ACA obtained for the samples grown for 15, 30, 45, and 60 min are 75.5 ± 3.8, 95.5 ± 6.9, 95.6 ± 9.8, and 90.8 ± 7.9°, respectively, while the ACA measured for the GaN substrate is 71.1 ± 2.6°.

The ACA of the porous as-grown GaN porous layers was higher than the intrinsic contact angle of GaN. This can be explained only by the Cassie–Baxter model, not by the Wenzel model (Supporting Information), because air is trapped between the pore cavities, creating a balance between the capillary force, which governs the penetration of water inside the pore, and the pressure of the trapped air, which acts against the penetration of the water into the pore.

**Figure 5:**
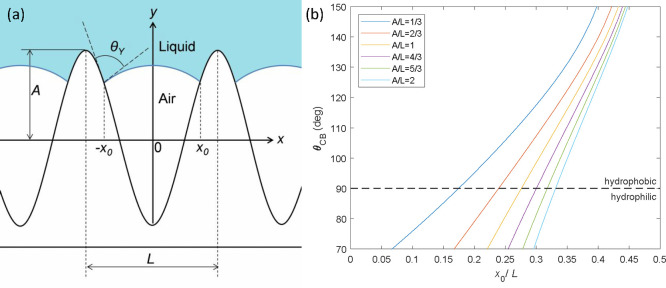
(a) Scheme of the Cassie–Baxter model for a sinusoidal microstructure proposed by Liu et al.^26^ (b) Apparent contact angles described by the Cassie–Baxter model (*θ*_CB_) considering a sinusoidal microstructure as a function of the wavelength-normalized triple-point position (*x*_0_/*L*) for different equispaced geometric values (*A*/*L*).

To fully understand the wetting mechanism in our samples, we considered the model proposed by Liu et al.^26^ (Figure 5a) in which the microstructure is simplified to a sinusoidal-like surface with a shape function *y*(*x*) = −*A* cos(*kx*), where *A* is the roughness amplitude, *k* is the wavenumber *k* = 2π/*L*, and *L* is the peak-to-peak distance. We also assumed that the liquid/gas interface within the microstructure has a circular arc shape with a radius *R*. The Young equation has to be satisfied locally at the triple contact point inside the microstructure (*x*_0_); therefore, the liquid/gas interface for a hydrophilic material has a convex meniscus *R* < 0 in the microstructure. This generates a negative Laplace pressure Δ*p* across the liquid/gas interface as described by the equation
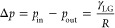
5where *p*_in_ is the pressure inside the liquid droplet and *p*_out_ is the pressure of the gas phase trapped in the microstructure.

When the geometric limitations and the energy analysis are taken into account, the Cassie–Baxter contact angle for a sinusoidal structure using Liu’s model^26^ is described by
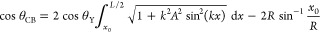
6

Using this model, we have plotted the Cassie–Baxter angle, θ_CB_, versus the dimensionless tripe-point position *x̃*_0_ = *x*_0_/*L* for different geometric values expressed by the dimensionless amplitude *Ã* = *A*/*L* (Figure 5b) using the value of the contact angle measured for the GaN substrate as the Young angle, *θ*_y_. With this, we are trying to model porous GaN surfaces with different degrees of porosity. As this wavelength, we take the sum of the mean pore size and the wall thickness measured by AFM. Nevertheless, due to the difficulty in measuring the pore depth, we cannot assign an *A* value to our samples, but we can use extreme values such as the overestimated value given by the cross-sectional SEM and the underestimated value given by the AFM profile. This would give us a range of *Ã* of between 0.3 and 1.25 for the sample grown for 30 min, and we expect to have a higher upper limit for samples grown for 45 and 60 min since they are thicker and have comparable *L* values. On the basis of the estimated range of *Ã*, the simulation for *θ*_CB_ against *x̃*_0_ is done over the range of 1/3 < *Ã* < 2. By applying this simulation, we observed that, even for the same geometric parameters, we can obtain a wide range of *θ*_CB_ depending on the position of the triple contact point, which is a direct consequence of how much air is trapped in the microstructure. Therefore, well-trapped air pockets in the microstructure will produce more hydrophobic surfaces. Moreover, we can see that hydrophobicity can be easily achieved for any *A*/*L* ratio, and even a superhydrophobic surface (ACA > 150°) could be generated if the right conditions are satisfied. It is worth mentioning that for higher *Ã* values the slope gets steeper and the curves of the equispaced *Ã* values are closer together, which means that at a certain point tuning the geometric parameters *Ã* will not produce a great change in the ACA. Instead, small changes in the triple contact point are key to tuning the ACA.

The wide range of pore diameters in the same sample could explain the deviation observed in the ACA of the porous GaN layers due to the constant change in *Ã* within a sample. In addition, the constant change in geometrical parameters *Ã* may lead to some pores being connected, giving rise to less-efficient air trapping in the microstructure changing the *x̃*_0_ value, explaining the observed difference in ACA in a sample.

## 4 Conclusions

Porous GaN epitaxial layers grown by CVD exhibit a larger apparent contact angle when compared to the flat nonporous GaN substrates. The apparent contact angle tends to increase until a hydrophobic regime is reached for the samples grown for deposition times longer than 30 min, reaching its higher value for the sample grown for 45 min, and then slowly decreases again as the deposition time increases, becoming a nearly hydrophilic material again for the samples grown for 60 min. According to these results and the Cassie–Baxter model for sinusoidal microstructures, it seems that the transformation toward a hydrophobic material is a compromise between the *A*/*L* ratio and how well air is trapped in the microstructure.

## Supporting Information Available

GaN nanoparticles’ XRD; model and figure of the apparent contact angle predicted by the Wenzel model on a GaN sinusodial surface (PDF)

## References

[ref1] Millán J.; Godignon P.; Perpiñà X.; Pérez-Tomás A.; Rebollo J. A Survey of Wide Bandgap Power Semiconductor Devices. IEEE Trans. Power Electron. 2014, 29 (5), 2155–2163. 10.1109/TPEL.2013.2268900.

[ref2] Saito W.; Takada Y.; Kuraguchi M.; Tsuda K.; Omura I. Recessed-gate structure approach toward normally off high-Voltage AlGaN/GaN HEMT for power electronics applications. IEEE Trans. Electron Devices 2006, 53 (2), 356–362. 10.1109/TED.2005.862708.

[ref3] Kim H.; Ohta J.; Ueno K.; Kobayashi A.; Morita M.; Tokumoto Y.; Fujioka H. Fabrication of full-color GaN-based light-emitting diodes on nearly lattice-matched flexible metal foils. Sci. Rep. 2017, 7 (1), 2112 10.1038/s41598-017-02431-7. 28522838 PMC5437013

[ref4] Nakamura S. The Roles of Structural Imperfections in InGaN-Based Blue Light-Emitting Diodes and Laser Diodes. Science 1998, 281 (5379), 956–961. 10.1126/science.281.5379.956. 9703504

[ref5] Schalwig J.; Müller G.; Eickhoff M.; Ambacher O.; Stutzmann M. Gas sensitive GaN/AlGaN-heterostructures. Sens. Actuators, B 2002, 87 (3), 425–430. 10.1016/S0925-4005(02)00292-7.

[ref6] Mehandru R.; Luo B.; Kang B. S.; Kim J.; Ren F.; Pearton S. J.; Pan C. C.; Chen G. T.; Chyi J. I. AlGaN/GaN HEMT based liquid sensors. Solid-State Electron. 2004, 48 (2), 351–353. 10.1016/S0038-1101(03)00318-6.

[ref7] Pearton S. J.; Kang B. S.; Kim S.; Ren F.; Gila B. P.; Abernathy C. R.; Lin J.; Chu S. N. G. GaN-based diodes and transistors for chemical, gas, biological and pressure sensing. J. Phys.: Condens. Matter 2004, 16 (29), R961–R994. 10.1088/0953-8984/16/29/R02.

[ref8] Li J.-d.; Cheng J.-j.; Miao B.; Wei X.-w.; Xie J.; Zhang J.-c.; Zhang Z.-q.; Wu D.-m. Detection of prostate-specific antigen with biomolecule-gated AlGaN/GaN high electron mobility transistors. J. Micromech. Microeng. 2014, 24 (7), 075023 10.1088/0960-1317/24/7/075023.

[ref9] Jewett S. A.; Makowski M. S.; Andrews B.; Manfra M. J.; Ivanisevic A. Gallium nitride is biocompatible and non-toxic before and after functionalization with peptides. Acta Biomater. 2012, 8 (2), 728–733. 10.1016/j.actbio.2011.09.038. 22019517

[ref10] Arisio C.; Cassou C. A.; Lieberman M. Loss of Siloxane Monolayers from GaN Surfaces in Water. Langmuir 2013, 29 (17), 5145–5149. 10.1021/la400849j. 23534848

[ref11] Wenzel R. N. Resistance of Solid Surfaces to Wetting by Water. Ind. Eng. Chem. 1936, 28 (8), 988–994. 10.1021/ie50320a024.

[ref12] Cassie A. B. D.; Baxter S. Wettability of porous surfaces. Trans. Faraday Soc. 1944, 40 (0), 546–551. 10.1039/tf9444000546.

[ref13] Mynbaeva M.; Titkov A.; Kryganovskii A.; Ratnikov V.; Mynbaev K.; Huhtinen H.; Laiho R.; Dmitriev V. Structural characterization and strain relaxation in porous GaN layers. Appl. Phys. Lett. 2000, 76 (9), 1113–1115. 10.1063/1.125955.

[ref14] Vajpeyi A. P.; Chua S. J.; Tripathy S.; Fitzgerald E. A.; Liu W.; Chen P.; Wang L. S. High Optical Quality Nanoporous GaN Prepared by Photoelectrochemical Etching. Electrochem. Solid-State Lett. 2005, 8 (4), G85 10.1149/1.1861037.

[ref15] Dıaz D. J.; Williamson T. L.; Adesida I.; Bohn P. W.; Molnar R. J. Morphology evolution and luminescence properties of porous GaN generated via Pt-assisted electroless etching of hydride vapor phase epitaxy GaN on sapphire. J. Appl. Phys. 2003, 94 (12), 7526–7534. 10.1063/1.1628833.

[ref16] Mahmood A.; Ahmed N. M.; Yusof Y.; Kwong Y. F.; Siang C. L.; Abd H. R.; Hassan Z. A Novel AC technique for high quality porous GaN. Int. J. Electrochem. Sci. 2013, 8 (4), 5801–5809.

[ref17] Mynbaeva M.; Bazhenov N.; Mynbaev K.; Evstropov V.; Saddow S. E.; Koshka Y.; Melnik Y. Photoconductivity in Porous GaN Layers. Phys. Status Solidi B 2001, 228 (2), 589–592. 10.1002/1521-3951(200111)228:2<589::AID-PSSB589>3.0.CO;2-J.

[ref18] Li X.; Kim Y.-W.; Bohn P. W.; Adesida I. In-plane bandgap control in porous GaN through electroless wet chemical etching. Appl. Phys. Lett. 2002, 80 (6), 980–982. 10.1063/1.1448860.

[ref19] Wang R.; Liu D.; Zuo Z.; Yu Q.; Feng Z.; Xu X. Metal-assisted electroless fabrication of nanoporous p-GaN for increasing the light extraction efficiency of light emitting diodes. AIP Adv. 2012, 2 (1), 012109 10.1063/1.3679150.

[ref20] Bilousov O. V.; Carvajal J. J.; Mena J.; Martínez O.; Jiménez J.; Geaney H.; Díaz F.; Aguiló M.; O’Dwyer C. Epitaxial growth of (0001) oriented porous GaN layers by chemical vapour deposition. CrystEngComm 2014, 16 (44), 10255–10261. 10.1039/C4CE01339E.

[ref21] Carvajal J. J.; Bilousov O. V.; Drouin D.; Aguiló M.; Díaz F.; Rojo J. C. Chemical Vapor Deposition of Porous GaN Particles on Silicon. Microsc. Microanal. 2012, 18 (4), 905–911. 10.1017/S1431927612001134. 22831653

[ref22] Morkoç H. General Properties of Nitrides. In Nitride Semiconductors and Devices; Morkoç H., Ed.; Springer: Berlin, 1999; pp 8–44.

[ref23] Mena J.; Carvajal J. J; Martinez O.; Jimenez J.; Zubialevich V. Z; Parbrook P. J; Diaz F.; Aguilo M. Optical and structural characterisation of epitaxial nanoporous GaN grown by CVD. Nanotechnology 2017, 28 (37), 375701 10.1088/1361-6528/aa7e9d. 28691692

[ref24] Miwa M.; Nakajima A.; Fujishima A.; Hashimoto K.; Watanabe T. Effects of the Surface Roughness on Sliding Angles of Water Droplets on Superhydrophobic Surfaces. Langmuir 2000, 16 (13), 5754–5760. 10.1021/la991660o.

[ref25] Liu J.; Mei Y.; Xia R. A New Wetting Mechanism Based upon Triple Contact Line Pinning. Langmuir 2011, 27 (1), 196–200. 10.1021/la103652s. 21117687

[ref26] Liu J.-L.; Feng X.-Q.; Wang G.; Yu S.-W. Mechanisms of superhydrophobicity on hydrophilic substrates. J. Phys.: Condens. Matter 2007, 19 (35), 356002 10.1088/0953-8984/19/35/356002.

