# Supplementary material for: Tailoring Wettability Properties of GaN Epitaxial Layers through Surface Porosity Induced during CVD Deposition
Source: Langmuir. 2021 Dec 10;37(50):14622–7. doi: 10.1021/acs.langmuir.1c02316 (PMC8697560; doi:10.1021/acs.langmuir.1c02316)
Supplement: Supplementary file 1 — la1c02316_si_001.pdf [file la1c02316_si_001.pdf]

Supporting information

Tailoring wettability properties of GaN epitaxial layers through surface porosity induced during CVD deposition

Josué Mena[1,2,*], Joan J. Carvajal[1], Vitaly Zubialevich[3], Peter J. Parbrook[3,4], Francesc Díaz[1], Magdalena Aguiló[1]

[1]Física i Cristal·lografia de Materials i Nanomaterials (FiCMA-FiCNA) and EMaS, Departament Química Física i Inorgànica, Universitat Rovira i Virgili (URV), Tarragona 43007, Spain

[2]Department of Physics, Umeå University, Umeå SE-90187, Sweden

[3]Tyndall National Institute, Lee Maltings, Cork T12 R5CP, Ireland

[4]School of Engineering, University College Cork, Cork T12 R5CP, Ireland

*E-mail: josue.mena@umu.se

Table of contents :

- Supporting Text. Wenzel contact angle in a sinusoidal structure
- Figure S1 The aparent contact angle predicted by the Wenzel model
- Figusre S2 Debye rings and X-ray difraction patterns of the nanoporous GaN film

Supporting Text. Wenzel contact angle in a sinusoidal structure

The predicted Wenzel contact angle based on the energetic analysis for a sinusoidal structure proposed by Liu *et al.*is described by:

$$\cos\theta_{\mathrm{W}} = 2\cos\theta_{\mathrm{Y}} \int_{0}^{L/2} \sqrt{1 + k^2 A^2 \sin^2(kx)}\, dx$$

where θ_{W} is the macroscopic Wenzel contact angle, θ_{Y} is the Young contact angle, A is the roughness amplitude, k is the wavenumber $k = 2\pi/L$, and L is the peak-to-peak distance.

The apparent contact angle (ACA) predicted using the Wenzel model (θ_W) has a maximum value of θ_{Y} when the dimensionless amplitude $\tilde{A} = A/L$ is 0, corresponding to a flat surface, and decreases as the $\tilde{A}$ increases as shown in Figure S1. Due to the fact that the ACA is always lower than the θ_{Y}, the Wenzel model cannot describe the wetting mechanism in the GaN porous structures, proving that the water does not fully penetrate the pores, wetting the hole surface, instead, a composite with air trapped between water and GaN surface is formed.

Supporting Figures.

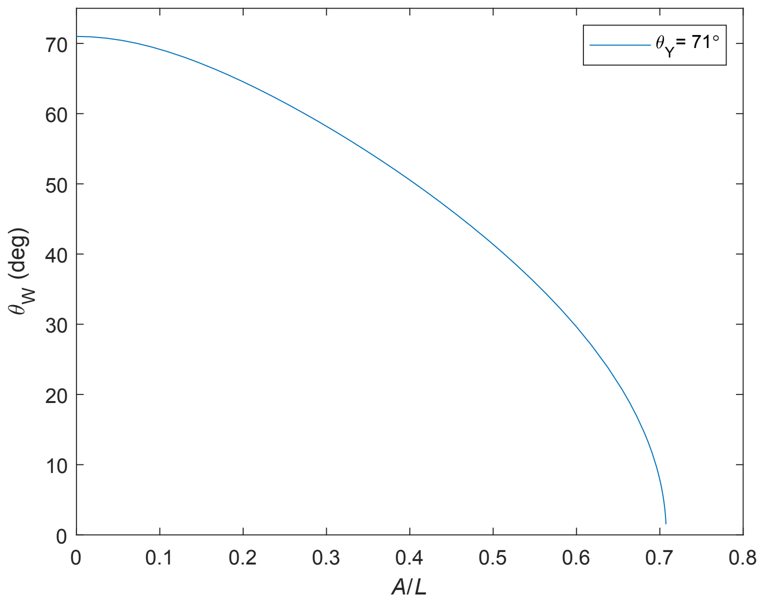

Figure S1. The apparent contact angle predicted by the Wenzel model as a function of the dimensionless amplitude, where we use $\theta_{\mathrm{Y}} = 71°$.

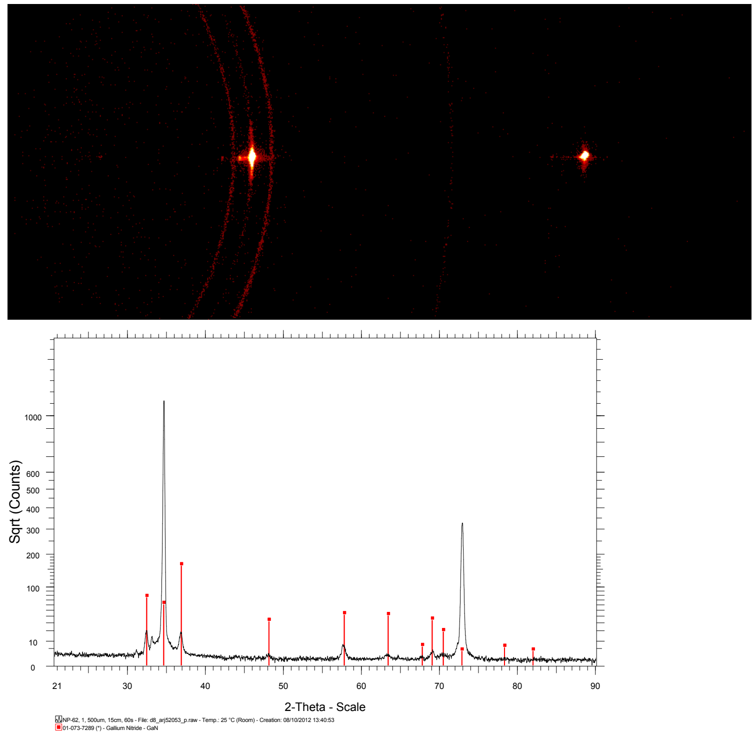

Figure S2. Debye rings and X-ray difraction patterns of the nanoporous GaN film deposited on 1 µm thick p-type GaN (0001) doped with Mg / 3 µm thick undoped GaN (0001) / sapphire (0001).
